# Two Methods of Nursing Insured Persons

**Published:** 1921-06-04

**Authors:** 


### Two Methods of Nursing Insured Persons.

Approved Societies seem to be at the parting of the ways in the matter of making provision for nursing their members. As our readers are aware, the National Insurance Act contains a section under which a nursing scheme can be started. Manchester has led the way, Preston and Leicester have followed on, and Hull has recently held a conference with a view to formulating a scheme for the same purpose. There are about 120,000 insured persons in Hull, and it is suggested that a contribution of 3d. per head per annum should be paid by the Societies, representing £1,250 to £1,500, to form a fund for paying the required nursing staff. As the local Jubilee Nursing Association is willing to co-operate we trust it may be possible to avoid the objectionable plan of engaging nurses solely for the care of insured persons, under which overlapping and consequent waste of time and money are inevitable.

The Scottish Branch of the Jubilee Institute has formulated a very interesting scheme for the nursing of insured persons. The Council are prepared to undertake this branch of nursing either on an annual *per capita* basis of payment, calculated at 4d. for each member of a Society, or at a charge of 3s. 6d. for the first three visits, 1s. 4d. per visit up to thirty-three visits, and 5s. a week thereafter, these rates being considered an equivalent of the *per capita* payment, though it is possible they may cost 1d. per head per annum more. Under the first system Approved Societies have little control, whereas under the second they have a general supervision of the working of the system through their representatives. In either case the services of a nurse can be requisitioned from the nursing headquarters as required, the nurse furnishing weekly or fortnightly reports to the Society. The first Friendly Society to adopt the above scale of fixed rates per visit is the British Order of Ancient Free Gardeners, to whom honour is due. The Isle of Wight County Nursing Association has lately adopted a scheme under which the rates are slightly lower.

There can be little doubt that the nursing of insured members of their Societies will shortly become universal. Apart from the general desire for nursing benefits for the relief of suffering, the Societies are beginning to recognise the fact that the best way of shortening an illness and getting an insured person back to work is to provide him with a nurse. Before long the nurses will very probably save the Societies as much as they cost. We doubt very much if they can be adequately paid on a *per capita* basis of threepence.

